# Geographic variation in opsin expression does not align with opsin genotype in Lake Victoria cichlid populations

**DOI:** 10.1002/ece3.5411

**Published:** 2019-07-09

**Authors:** Daniel Shane Wright, Roy Meijer, Roel van Eijk, Wicher Vos, Ole Seehausen, Martine E. Maan

**Affiliations:** ^1^ Groningen Institute for Evolutionary Life Sciences (GELIFES) University of Groningen Groningen The Netherlands; ^2^ University of Applied Sciences van Hall Larenstein Leeuwarden The Netherlands; ^3^ Institute of Ecology & Evolution University of Bern Bern Switzerland; ^4^ Department Fish Ecology & Evolution Eawag, Center for Ecology, Evolution and Biogeochemistry Kastanienbaum Switzerland

**Keywords:** ecological speciation, haplochromine, LWS, sensory drive

## Abstract

Sensory adaptation to the local environment can contribute to speciation. Aquatic environments are well suited for studying this process: The natural attenuation of light through water results in heterogeneous light environments, to which vision‐dependent species must adapt for communication and survival. Here, we study visual adaptation in sympatric *Pundamilia* cichlids from southeastern Lake Victoria. Species with blue or red male nuptial coloration co‐occur at many rocky islands but tend to be depth‐differentiated, entailing different visual habitats, more strongly at some islands than others. Divergent visual adaptation to these environments has been implicated as a major factor in the divergence of *P. pundamilia* and *P. nyererei*, as they show consistent differentiation in the long‐wavelength‐sensitive visual pigment gene sequence (LWS opsin). In addition to sequence variation, variation in the opsin gene expression levels may contribute to visual adaptation. We characterized opsin gene expression and LWS genotype across *Pundamilia* populations inhabiting turbid and clear waters, to examine how different mechanisms of visual tuning contribute to visual adaptation. As predicted, the short‐wavelength‐sensitive opsin (SWS2b) was expressed exclusively in a population from clear water. Contrary to prediction however, expression levels of the other opsins were species‐ and island‐dependent and did not align with species differences in LWS genotype. Specifically, in two locations with turbid water, the shallow‐water dwelling blue species expressed more LWS and less RH2A than the deeper‐dwelling red species, while the opposite pattern occurred in the two locations with clear water. Visual modeling suggests that the observed distribution of opsin expression profiles and LWS genotypes does not maximize visual performance, implying the involvement of additional visual tuning mechanisms and/or incomplete adaptation.

**OPEN RESEARCH BADGE:**

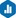

This article has earned an Open Data Badge for making publicly available the digitally‐shareable data necessary to reproduce the reported results. The data is available at https://hdl.handle.net/10411/I1IUUQ.

## 1 INTRODUCTION

Sensory adaptation to divergent environmental conditions can be consequential for speciation (Boughman, 2002), affecting both ecological performance and sexual communication. In aquatic systems, environmental light is strongly depth‐dependent and mediated by the physical properties of both the water itself and its dissolved and suspended content. For aquatic organisms reliant on vision for communication and survival, selection on the visual systems is often strong. Indeed, habitat‐associated visual adaptation has been documented in both freshwater (Bowmaker et al., 1994; Ehlman, Sandkam, Breden, & Sih, 2015; Fuller, Fleishman, Leal, Travis, & Loew, 2003; Veen, Brock, Rennison, & Bolnick, 2017) and marine environments (Lythgoe, Muntz, Partridge, Shand, & Williams, 1994; Partridge, Shand, Archer, Lythgoe, & Groningen‐Luyben, 1989; Shand et al., 2008; White, Goncalves, Partridge, & Oliveira, 2004). Here, we characterize variation in visual pigment gene sequence and expression in multiple populations of Lake Victoria cichlids (*Pundamilia* spp.), aiming to understand how these two visual system properties diverge across visual habitats.

In fish (and vertebrates in general), visual sensitivity is determined by photosensory pigments in the retina, comprised of a light‐sensitive chromophore bound to an opsin protein (Bowmaker, 1990). The haplochromine cichlids of East Africa possess seven distinct classes of opsins, each maximally sensitive to different wavelengths of light. The rod opsin (RH1) functions in low light, while cone opsins mediate color vision in bright light. The cichlid cone opsins include (Carleton et al., 2008): the short‐wavelength‐sensitive opsins: SWS1 (UV), SWS2b (violet), SWS2a (blue), the rhodopsin‐like opsins: RH2B, RH2Aβ & RH2Aα (green), and the long‐wavelength‐sensitive opsin: LWS (red). Typically, cichlids express a subset of three cone opsins at a time (Carleton, 2009), related to the visual conditions of the habitat (Carleton, 2009; Carleton & Kocher, 2001; Carleton, Parry, Bowmaker, Hunt, & Seehausen, 2005; Hofmann et al., 2009; Van der Meer & Bowmaker, 1995; Smith et al., 2011). For example, in clear water Lake Malawi, cichlid opsin expression falls into distinct profiles, correlated with species‐specific depth range and ecology (Carleton, 2009; Carleton, Dalton, Escobar‐Camacho, & Nandamuri, 2016): The “short” profile (SWS1, RH2B, RH2A) confers greater UV/short‐wavelength sensitivity, a “medium” profile confers more short to middle‐wavelength sensitivity (SWS2b, RH2B, RH2A), and a “long” profile (SWS2a, RH2A, LWS) confers greater long‐wavelength sensitivity. In Lake Victoria, where water conditions are much more turbid and the light environment shifted to longer wavelengths, the “long” profile dominates; all cichlids studied so far express SWS2a, RH2A, and LWS (and low amounts of SWS2b; Hofmann et al., 2009).

East African cichlids not only exhibit habitat‐associated variation in opsin expression, but also variation in the coding sequence of the opsin genes (Carleton, 2009; Carleton et al., 2005; Hofmann et al., 2009; Seehausen et al., 2008; Terai, Mayer, Klein, Tichy, & Okada, 2002; Terai et al., 2006). Both factors influence color vision, which in cichlids, has been shown to affect ecological performance (foraging; Jordan, Howe, Juanes, Stauffer, & Loew, 2004) and, as male color is important for female mate preference (Maan & Sefc, 2013), it may also influence mating patterns. Together, these observations suggest that color vision and adaptation to the local light environment play an important role in cichlid speciation (Maan & Seehausen, 2010, 2011; Seehausen, Alphen, & Witte, 1997).

**Figure 1:**
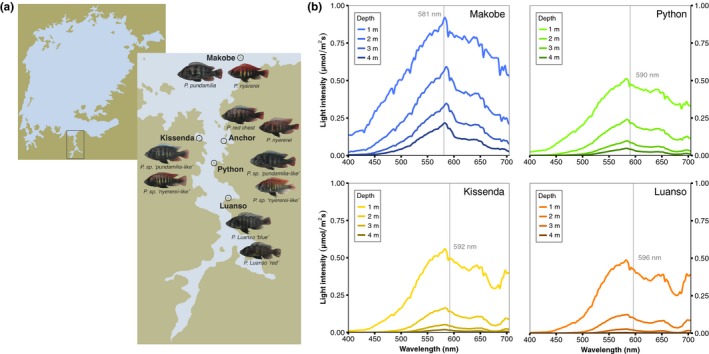
Sampling locations. (a) Blue and red *Pundamilia* males were sampled from five island locations in southeastern Lake Victoria. (b) Irradiance spectra at four of the sampling locations (irradiance was not measured at Anchor Island). Vertical lines indicate the spectral midpoint at 1 m depth: The wavelength at which the total light intensity of shorter wavelengths is equal to that of longer wavelengths

*Pundamilia pundamilia* (Seehausen, Lippitsch, Bouton, & Heleen, 1998) and *Pundamilia nyererei* (Witte‐Maas & Witte, 1985) form one of the best‐studied pairs of closely related rock‐dwelling haplochromine cichlids. They occur at rocky islands in southeastern Lake Victoria. Similar sympatric *Pundamilia* species pairs (*P. *sp.* *“*pundamilia‐like*” & *P. *sp.* *“*nyererei‐like*”) occur further south in the Mwanza Gulf (Meier et al., 2017; Meier, Marques, Wagner, Excoffier, & Seehausen, 2018; see Figure 1a). Males of the sympatric species are distinguished by their nuptial coloration; *P. pundamilia* and *P. *sp.* *“*pundamilia‐like*” are blue/gray, whereas *P. nyererei* and *P. *sp.* *“*nyererei‐like*” are bright orange or red dorsally and yellow on the flanks; all males have black vertical bars on the flanks. Females of both species are yellow/gray (Seehausen, 1996). At each location, the sympatric species tend to have different depth distributions—the blue species occur in shallow waters while the red species extends to greater depths (Seehausen, 1996; Seehausen et al., 2008). High turbidity in Lake Victoria results in a shift of the light spectrum toward longer wavelengths with increasing depth and, as such, the red species tend to inhabit an environment largely devoid of short‐wavelength light (Maan, Hofker, Alphen, & Seehausen, 2006; Seehausen et al., 2008; Castillo Cajas, Selz, Ripmeester, Seehausen, & Maan, 2012; see also Figure 1b). Previous work has shown that, in comparison with *P. pundamilia*, *P. nyererei* has greater behavioral sensitivity to long‐wavelength light (Maan et al., 2006). In line with this, both red species, *P. nyererei* and *P. *sp.* *“*nyererei‐like*,” carry LWS alleles that confer a more red‐shifted sensitivity, compared to the allele that dominates in *P. pundamilia* and *P. *sp.* *“*pundamilia‐like*” (Carleton et al., 2005; Seehausen et al., 2008).

Information on opsin expression in *Pundamilia* is limited and inconsistent. To date, measurements are based on a few, lab‐bred individuals, originating from different locations (Carleton et al., 2005; Hofmann et al., 2009), with different evolutionary histories (Meier et al., 2018, 2017). Moreover, given that levels of opsin expression are subject to phenotypic plasticity (Fuller, Carleton, Fadool, Spady, & Travis, 2005; Shand et al., 2008; Hofmann, O'Quin, Smith, & Carleton, 2010; Smith, Ma, Soares, & Carleton, 2012; Sakai, Ohtsuki, Kasagi, Kawamura, & Kawata, 2016; Veen et al., 2017; Wright, D.S., van Eijk, R., Schuart, L., Seehausen, O., Groothuis, T.G.G., Maan, M.E. *in prep*), laboratory‐housed fish may have different expression levels than those sampled in the natural habitat. Thus, to establish how variation in opsin expression contributes to divergent visual adaptation, it is necessary to determine expression patterns in a representative sample of wild fish, from both species and from multiple locations.

In this study, we characterize the opsin expression profiles of wild caught blue and red *Pundamilia* from multiple islands in southeastern Lake Victoria (Figure 1a). Based on the findings of Hofmann et al. (2009; higher SWS2b expression in Lake Victoria cichlids from short‐wavelength rich environments), we predict that SWS2b expression will be highest at locations with relatively clear waters, where short‐wavelength light penetrates deeper than in more turbid locations (Hofmann et al., 2009; Maan et al., 2006; Seehausen et al., 1997). Within island locations, we predict that LWS expression will be highest in the red species and SWS2b/SWS2a expression will be highest in the blue species, in line with their respective visual habitats and with observations in laboratory fish (Carleton et al., 2005). In cichlids, the cone opsins are arranged in a retinal mosaic: single cones expressing SWS1 or SWS2 are surrounded by double cones expressing RH2 and/or LWS (Carleton et al., 2016). As RH2A and LWS are both expressed in double cones, an increase in one means a decrease in the other. Thus, we also predict that RH2A expression will be higher in the blue species. Finally, we quantify whether variation in opsin expression, within and among islands, covaries with variation in the visual environment and LWS genotype, and we use visual modeling to evaluate whether the observed patterns of opsin expression and genotype are adaptive.

## 2 METHODS

### 2.1 Fish

In 2014 (September–November), we sampled *Pundamilia* of both sympatric species/morphs from five rocky islands in the open lake and Mwanza Gulf of Lake Victoria (Figure 1a). We included only males, as they could be easily identified from physical characteristics (morphology, color), whereas females are cryptically colored and hard to identify (Seehausen, 1996). For clarity, we refer to the differently colored males as *species* at all locations except Luanso Island (more details below), *island* denotes our different sampling locations, and *population* indicates species‐island combinations. We sampled males from Luanso (2°41′20.04″S, 32°53′3.12″E), Kissenda (2°32′57.84″S, 32°49′39.36″E), Python (2°37′25.68″S, 32°51′23.76″E), Anchor (2°33′18.72″S, 32°53′5.28″E), and Makobe Islands (2°21′55.44″S, 32°55′22.08″E). These locations represent a turbidity transect, with differing light conditions—on the south end is turbid water Luanso Island (Secchi disk: ~50 cm) and on the north end is clear water Makobe Island (Secchi: >200 cm). In total, we collected 112 males (Luanso = 29, Kissenda = 28, Python = 22, Anchor = 11, Makobe = 22);

Until population genomic analyses permitted a detailed understanding of the evolutionary and demographic history of this species group, all red populations were thought to belong to *P. nyererei* and all blue populations to *P. pundamilia*. However, the populations at Python and Kissenda Islands represent a separate speciation event, where similar blue “*pundamilia‐like*” and red “*nyererei‐like*” species have arisen after hybridization of *P. pundamilia* and *P. nyererei* from open water locations (i.e. Makobe Island; Meier et al., 2017, 2018). Importantly though, the alleles at the LWS, SWS2a, and SWS2b loci in the species pair *P. pundamilia * /*P. nyererei* and *P. *sp. “*pundamilia‐like*” / *P. *sp. “*nyererei‐like*” are the same (Meier et al., 2018).

The blue species at Anchor Island has not previously been studied. It is referred to as *Pundamilia *“*red chest*” (Seehausen, 1996) and resembles the other blue species in ecology (occupying shallow habitat around ~1–2 m depth) and morphology, but males have orange‐red coloration on the operculum and behind the pelvic fin (Seehausen, 1996). At Luanso, finally, blue and red phenotypes show no detectable genetic differentiation (Meier et al., 2018; Seehausen et al., 2008) and we categorized individuals as blue, intermediate, or red by visually scoring coloration. As in previous studies, we used the mean color scores of multiple observers (Dijkstra, Hekman, Hekman, Schulz, & Groothuis, 2007; Dijkstra, Seehausen, Seehausen, Pierotti, & Groothuis, 2007; Seehausen et al., 2008). Intermediate phenotypes at other locations (Python and Kissenda) occur at very low frequencies and were not included in the analyses (see Supplemental Information for opsin expression data of a few Python and Kissenda individuals morphologically categorized as “intermediate”).

**Figure 2:**
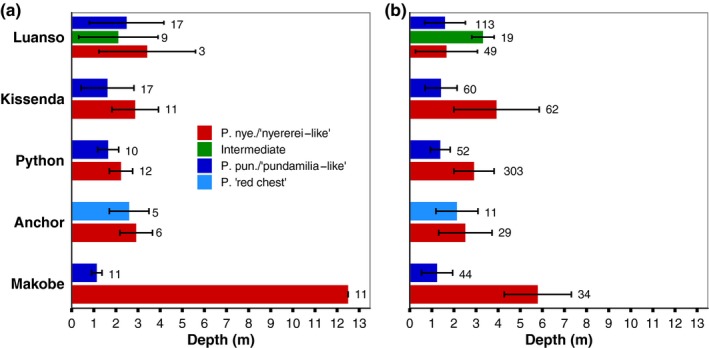
Depth distribution of sampled fish. (a) Mean capture depth (± standard deviation) of fish used in this study compared to the (b) depth distributions reported by Seehausen et al. (2008; Anchor Island depth distributions are from unpublished field data collected by OS in 1991/1992). Sample sizes are indicated above each bar

At each island, fish were caught by angling and gill netting, noting capture depth (Figure 2a), and held in keep nets at ~1 m depth. All fish were sampled within the depth ranges reported by Seehausen et al. (2008), expect for Makobe *P. nyererei*, which was sampled deeper (Figure 2). At Makobe, *P. nyererei* are abundant at depths >5 m (DSW, OS, MEM, personal observations), so our deeper sampling is still within the species natural depth range. In the late afternoon, the fish were transported alive to the Tanzania Fisheries Research Institute (TAFIRI ‐ Mwanza Centre), where they were euthanized with 2‐Phenoxyethanol (~2.5 ml/L) and the eyes extracted, preserved in *RNAlater*™ (Ambion), and frozen. Later, the samples were shipped to the University of Groningen, the Netherlands for analyses. To maximize RNA yield and minimize differences due to circadian variation in opsin expression (Halstenberg et al., 2005), all fish were euthanized in the early evening on the day of capture (~17:00–20:00). Though the time between fish capture and eye sampling was variable (most were captured in the early afternoon, but some were caught later, ~16:00), we do not expect this to have influenced the patterns we report here; recent work in guppies suggests that light‐induced changes in the visual system occur over longer time periods (>24 hr; Cole, Lynn, Kranz, & Endler, 2019). Sampling was conducted with permission of the Tanzania Commission for Science and Technology (COSTECH—No. 2013‐253‐NA‐2014‐117).

### 2.2 Opsin mRNA expression

We used real‐time polymerase chain reaction (qPCR) to determine the relative amount of each cone opsin gene expressed (Carleton et al., 2005). From preserved eyes, we removed the retina and isolated total RNA using Trizol (Ambion). We reverse transcribed one microgram of total RNA using Oligo(dT)_18_ primer (Thermo Scientific) and RevertAid H Minus Reverse Transcriptase (Thermo Scientific) at 45°C to create retinal cDNA. qPCR reactions were set up for each cone opsin (SWS2b, SWS2a, RH2A, LWS) using TaqMan chemistry (Applied Biosystems) and gene‐specific primers and probes (Table S1). As in previous studies, we collectively measured the functionally and genetically similar RH2Aα and RH2Aβ as RH2A (Carleton et al., 2005, 2008; Hofmann et al., 2009; Spady et al., 2006). Fluorescence was monitored with a CFX96 Real‐Time PCR Detection System (Bio‐Rad) over 50 cycles (95°C for 2 min; 95°C for 15 s; 60°C for 1 min).

We used *LinRegPCR* (Ramakers, Ruijter, Deprez, & Moorman, 2003) to determine the critical threshold cycle numbers (*C_t_*) for all four opsin genes. This approach examines the log‐linear part of the PCR curve for each sample, determining the upper and lower limits of a “window‐of‐linearity” (Ramakers et al., 2003). Linear regression analysis can then be used to calculate the individual PCR efficiency and to estimate the initial concentration (*N*_0_) from a line that best fits the data (Ramakers et al., 2003). In this way, *N*_0_ values can be estimated without having to assume equal PCR efficiencies between amplicons (Ramakers et al., 2003). All samples were run in duplicate and for consistency, we applied specific quality control parameters: PCR efficiency 75%–125% and *Ct* standard deviation ≤ 0.5. We used the mean of the duplicate *N*_0_ estimates to calculate relative expression levels for each sample (described below).

On each plate, we included a serially diluted construct containing one fragment of each of the four opsin genes ligated together. From this, we used linear regression to examine the relationship between Log (concentration) and *Ct* values of the construct, enabling us to calculate the slope (*m*) and intercept (*b*) of the regression. Using these values, we calculated relative cone opsin expression as:

$\frac{N_{0i}}{N_{0\mathrm{all}}}=\frac{{\text{exp}}^{\frac{\left(Ct_{i}-b\right)}{m}}}{\sum {\text{exp}}^{\frac{\left(Ct_{i}-b\right)}{m}}}$

where *N_0i_/N*_0all_ is the expression for a given opsin gene relative to the total expression of all measured opsin genes, *Ct_i_* is the critical threshold value for the focal sample, and *b* and *m* are the intercepts and slope values derived from the construct linear regression (as detailed in Gallup, 2011). This approach differs from previous work on *Pundamilia* opsin expression, where only the slope (efficiency) of the construct was considered (Carleton et al., 2005: see Supplemental Information for details of both approaches).

### 2.3 LWS sequence variation

LWS is the most variable visual pigment among Lake Victoria cichlids (Spady et al., 2005; Terai et al., 2002), and there is evidence for strong parallel divergent selection on LWS, both between *P. pundamilia* and *P. nyererei* and between *P. *sp. “*pundamilia‐like*” and* P. *sp. “*nyererei‐like*” (Meier et al., 2018; Seehausen et al., 2008). *Pundamilia* harbors three forms of the LWS gene: The “H” allele, with peak sensitivity at 559 ± 1 nm, the “P” allele, with peak sensitivity at 544 ± 3 nm, and the “M3” allele, considered a recombinant form (Seehausen et al., 2008). The “H” allele occurs predominantly in red phenotypes—in *P. nyererei* at Makobe and *P. *sp. “*nyererei‐like*” at Python and Kissenda, while the “P” allele occurs predominantly in blue phenotypes—in *P. pundamilia* at Makobe and *P. *sp. “*pundamilia‐like*” at Python and Kissenda (Seehausen et al., 2008). The alleles differ in only three amino acid positions (216, 230, 275), located on the fourth and fifth exons (Seehausen et al., 2008; Terai et al., 2006).

From fin clips, we isolated DNA (Meeker, Hutchinson, Ho, & Trede, 2007) and sequenced the LWS gene of 90 fish that were also measured for opsin expression (*Sanger* sequencing, GATC Biotech). We sequenced exons 4 and 5 (498 bp, including the 91 bp intron; Forward primer: GTTTGGTGTGCTCCTCCCAT; Reverse primer: CAGAGCCATCGTCCACCTGT: Figure S1) and categorized individuals as “H” if: 216Y, 230A, 275C, “P” if: 216F, 230T, 275I, and “M3” if: 216Y, 230T, 275I (as in Seehausen et al., 2008). Isolation of genomic DNA was unsuccessful for 21 fish, so we sequenced their cDNA generated for qPCR (see above). For 11 individuals (nonheterozygous, see below), we sequenced both gDNA and cDNA to establish that sequencing results of both methods were identical (see Table S2). All fish were sequenced twice, in forward and reverse directions, and alignments were performed in Mega 7 (Kumar, Stecher, & Tamura, 2016), using the LWS coding sequences reported in Seehausen et al. (2008) as reference. For 17 fish, we observed multiple peaks at one or more of the polymorphic nucleotide sites (see Figure S2). We had gDNA and cDNA for 11 of these individuals; heterozygosity was confirmed by sequencing both sample types (Table S2). In total, we sequenced 111 of 112 fish measured for opsin expression (we failed to sequence one red male from Makobe Island).

### 2.4 Light measurements

We used the light measurements reported in Castillo Cajas et al. (2012), who measured downwelling irradiance (in μmol/(m^2^*s)) at each island (Figure 1b) using a BLK‐C‐100 spectrometer and F‐600‐UV‐VIS‐SR optical fiber with CR2 cosine receptor (Stellar‐Net, FL). Measurements were collected between 8:00 and 12:00 hr at 0.5 m depth increments, starting at 0.5 m depth and going down until approximately 6 m (deeper at less turbid locations). In 2010, two independent measurement series were collected from Luanso (29 May, 7 June), three from Kissenda (17 May, 1 June, 9 June), and four from Python (20 May, 26 May, 4 June, 5 June) and Makobe Islands (22 May, 27 May, 3 June, 10 June). Irradiance measurements were not conducted at Anchor Island (see below). Within every measurement series, we averaged a minimum of two irradiance spectra for each depth and then took the mean of the depth measurements across sampling days (thus, the mean of two measurements at each depth and then average of means across multiple days).

Spectral measurements and fish collections were conducted separately (2010 vs. 2014), but we do not expect this to influence the results presented here. Prior work has shown consistency in (a) the differences in the light conditions between the sampling locations (as reported in Table 1 of Castillo Cajas et al., 2012: "*for Secchi readings collected during 2000–2010 at our four sampling sites: ANOVA controlling for sampling date: F*_3,107_ *= *25.41,* p *< 0.0001") and (b) the shape of the light spectra (Figure 3 of Seehausen et al. (1997) is consistent with Castillo Cajas et al. (2012) and Figure 1 of the present study). Thus, the differences in visual conditions between depths (at every location) is highly consistent and the direction of this difference does not change (i.e., deeper habitats will always have less short‐wavelength light).

**Table 1:** Opsin expression depends on location and species identity, but not the light environment

Fixed effect	SWS2b	SWS2a	RH2A	LWS
Species*island	*χ* ^2^(4) = 4.70, *p* = 0.31	*χ* ^2^(4) = 3.29, *p* = 0.50	***χ*^2^(4) = 39.9, *p* < 0.001**	***χ*^2^(4) = 21.6, *p* < 0.001**
OR (individual)	*χ* ^2^(1) = 0.17, *p* = 0.67	*χ* ^2^(1) = 0.32, *p* = 0.56	*χ* ^2^(1) = 0.66, *p* = 0.41	*χ* ^2^(1) = 0.55, *p* = 0.45
LWS genotype	*χ* ^2^(3) = 2.85, *p* = 0.41	*χ* ^2^(3) = 1.90, *p* = 0.59	***χ*^2^(3) = 18.7, *p* < 0.001**	***χ*^2^(3) = 13.2, *p* < 0.01**
Species	*χ* ^2^(2) = 4.03, *p* = 0.13	*χ* ^2^(2) = 2.88, *p* = 0.23	***χ*^2^(2) = 5.25, *p* = 0.072**	***χ*^2^(2) = 8.86, *p* = 0.011**
Island	***χ*^2^(4) = 94.4, *p* < 0.001**	***χ*^2^(4) = 12.4, *p* = 0.014**	***χ*^2^(4) = 9.79, *p* = 0.044**	***χ*^2^(4) = 33.5, *p* < 0.001**
OR (population)	***χ*^2^(1) = 7.88, *p* < 0.01**	*χ* ^2^(1) = 1.81, *p* = 0.17	*χ* ^2^(1) = 0.73, *p* = 0.39	*χ* ^2^(1) = 0.19, *p* = 0.65

Note

**Figure 3:**
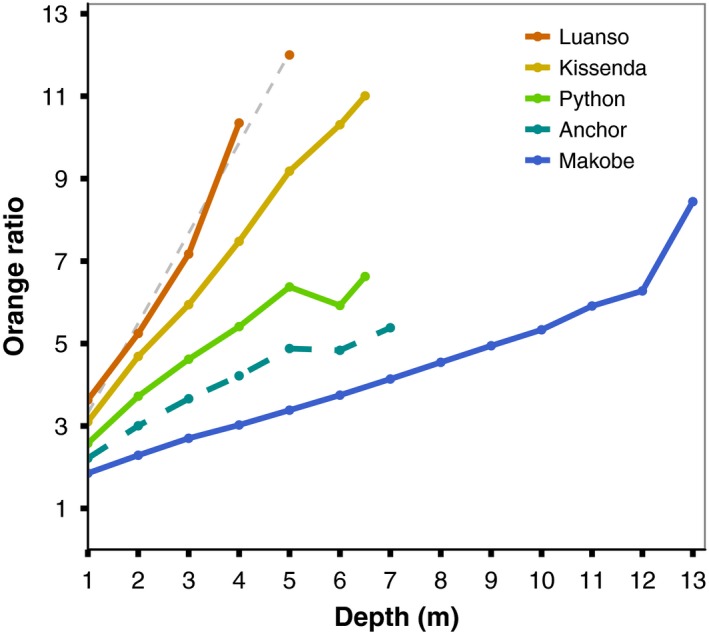
Light environments at the study locations. Orange ratio (OR) increases with depth at all islands; Luanso, the most turbid location (Secchi disk: ~50 cm), has the highest OR. Irradiance spectra for Anchor Island were unavailable, so OR values were estimated as the median of the OR values at Makobe and Python Islands. At Luanso Island, spectra were only available to 4 m depth, thus linear regression (gray dashed line) was used to estimate OR for fish captured at 5 m

**Figure 4:**
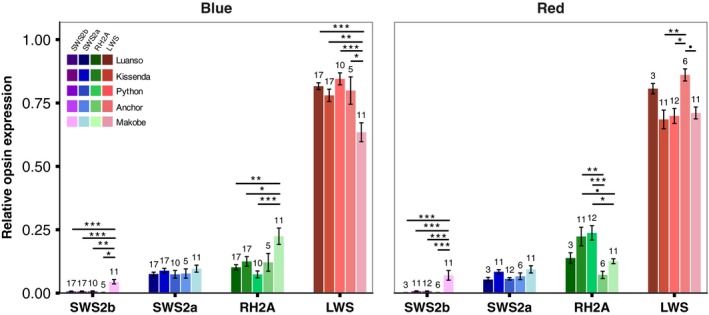
Geographic variation in opsin expression. The blue and red species expressed *SWS2b* at clear water Makobe Island only (blue: *p* < 0.01; red: *p* < 0.001). *SWS2a* expression was influenced only by the individual effect of island (*χ* ^2^(4) = 12.42, *p* = 0.014); for all fish (both species combined), expression at Makobe was higher than at Python (*Z* = 2.91, *p* = 0.028) and slightly higher than at Luanso (*Z* = 2.57, *p* = 0.074). *RH2A*: there was a significant island by species interaction (*χ* ^2^(4) = 39.96, *p* < 0.001). Tukey Post hoc revealed higher RH2A expression in Makobe blue phenotypes compared to Python, Kissenda, and Luanso (*p* < 0.029); Anchor and Makobe did not differ (*p* = 0.29). Makobe red phenotypes expressed less RH2A than red phenotypes at Python (*Z* = −3.50, *p* = 0.018) and slightly less than those at Kissenda (*Z* = −2.99, *p* = 0.090). RH2A expression in Anchor Island red types was lower than both Python and Kissenda (*p* < 0.01) but did not differ from Makobe (*p* = 0.9). *LWS*: again, there was a significant interaction between island and species (*χ* ^2^(4) = 21.62, *p* = 0.0002). Post hoc revealed lower LWS expression in Makobe blue phenotypes compared to all other locations (*p* < 0.038). For the red phenotypes, LWS expression was higher at Anchor (Anchor vs. Python: *Z* = 3.48, *p* = 0.02; Anchor vs. Kissenda: *Z* = 3.72, *p* < 0.01; Anchor vs. Makobe: *Z* = 3.18, *p* = 0.052). Sample sizes are indicated above each bar and error bars represent ± standard error. ***indicates *p* < 0.001, **indicates *p* < 0.01, *indicates *p* < 0.05, • indicates *p* < 0.1.

We examined how opsin expression was influenced by the interaction of species and island, as well as the light environment and LWS genotype. Orange ratio (OR), based on either individual capture depth or population‐level depth distribution, did not covary with opsin expression. The only exception was SWS2b, which displayed a significant, negative relationship with population‐level OR. However, this can be attributed to the fact that SWS2b was expressed at Makobe Island only (lowest OR; see Figure 3); at all other locations, SWS2b expression was essentially zero (with higher OR scores; see also Figure 4). Together, these results indicate that the light environment alone does not predict the observed variation in opsin expression. Significant effects are indicated in bold.

To quantify depth‐associated changes in visual conditions, we calculated the *orange ratio* (OR)—the ratio of light transmitted in the 550–700 nm range over the transmittance in the 400–549 nm range (Endler & Houde, 1995). *Pundamilia* do not express the SWS1 opsin and the peak sensitivity of the LWS opsin is ~565 nm (Carleton, 2009; Carleton et al., 2005), so visual sensitivity <400 nm or >700 nm is unlikely. We assigned OR values based on the capture depth of each fish. Since spectral measurements at Anchor Island were unavailable, we estimated OR as the median of the orange ratios observed at each depth for Makobe and Python Islands (Figure 3). This estimate was based on prior work showing that the water transparency (Bouton, Seehausen, & Alphen, 1997; Mrosso, Msuku, & Seehausen, 2004) and spectral width (Bouton, Visser, & Barel, 2002) at Anchor Island are intermediate to the other two locations. Additionally, the light spectrum at Anchor Island has a similar shape as that in other locations in the Mwanza Gulf (see Figure 3 in Seehausen et al., 1997). Spectral measurements at Luanso Island were only available down to 4 m depth (light intensity is too low in deeper waters), so we used linear regression to estimate OR experienced by fish caught deeper (~5 m).

### 2.5 Quantum catch

To evaluate whether population‐specific combinations of opsin expression and LWS genotype optimized light capture, we compared the estimated visual performance of local fish with that of hypothetical immigrants from all other locations. We calculated quantum catch—the amount of light captured by the visual system in a given light environment (Kelber, Vorobyev, & Osorio, 2003)—for both sympatric species at each location, except Anchor Island (no spectral measurements, see above). Quantum catch estimates were obtained for each opsin by multiplying relative expression of that opsin with the depth‐specific irradiance spectrum, assigned from the capture depth of each fish. Visual sensitivity in cichlids is also affected by differential chromophore usage (vitamin A1 vs. A2; Torres‐Dowdall et al., 2017) but this has never been measured in *Pundamilia*. Thus, our quantum catch estimates incorporated only LWS genotype and opsin expression, using previously reported peak sensitivity values for each opsin, from A1‐derived retinal (Carleton, 2009; Seehausen et al., 2008; Spady et al., 2006). We calculated quantum catch (*Q_c_*) as follows:

$Q_{\mathrm{ci}}=\int_{400\text{nm}}^{700\text{nm}}I\left(\lambda \right)R\left(\lambda \right)N_{i}$

where *I*(*λ*) is the island‐specific, normalized irradiance spectrum for each capture depth, *R(λ)* is the photoreceptor sensitivity (based on the equations of Govardovskii, Fyhrquist, Reuter, Kuzmin, & Donner, 2000), and *N_i_* is the relative opsin expression for each individual. Absorbance values were calculated separately for the two LWS alleles, H (*λ*_max_ = 559 nm) and P (*λ*_max_ = 544 nm; Seehausen et al., 2008), while *λ*_max_ for the other three opsins were constant: SWS2b (*λ*_max_ = 425 nm), SWS2a (*λ*_max_ = 455 nm), RH2A (*λ*_max_ = 528 nm); Spady et al., 2006; Carleton, 2009). For heterozygous LWS genotypes, we assumed an intermediate visual phenotype and calculated *Q_c_* based on the median sensitivity of the “H” and “P” alleles (*λ*_max_ = 551.5 nm). We excluded the two Kissenda Island fish with mismatched LWS genotypes (i.e., blue fish with the “HH” genotype). To compare the visual performance of residents to hypothetical immigrants, we calculated the frequency‐weighted mean depth for each genotype/island combination and “transplanted” the immigrants to that depth.

### 2.6 Statistical analysis

Prior to analyses, data were filtered for outliers, calculated as 1.5 * the interquartile range (IQR). This was done separately for each opsin/population combination, resulting in opsin expression data for 112 fish (17 samples did not pass the filter). We used this additional filtering step to ensure that all data were consistently within a natural range of expression values (as documented in Carleton et al., 2005; Hofmann et al., 2009; plus our own measurements of opsin expression in lab‐bred fish; Wright et al*. in prep*) and were not artifacts of the qPCR procedure and/or the sampling methodology. Using generalized linear modeling (GLM), we explored how opsin expression differed between populations and was influenced by OR and LWS genotype as follows: expression ~ species * island + OR + genotype. The significance of fixed effect parameters was determined by likelihood ratio tests (LRT) via the *drop1* function and minimum adequate statistical models were selected using statistical significance (Crawley, 2002; Nakagawa & Cuthill, 2007). We used the *ANOVA* function in the *car* package (Fox et al., 2017) to estimate the parameters of significant fixed effects. In the case of more than two categories per fixed effect parameter (i.e., islands), we used post hoc Tukey tests (glht—multcomp package: Hothorn, Bretz, & Westfall, 2008) to obtain parameter estimates and report *p*‐values adjusted for multiple comparisons.

## 3 RESULTS

Patterns of opsin expression differed significantly between islands and between species. Species differences were found at most islands, but the direction of differences between the blue and red phenotypes was not consistent between islands. We first present between‐island variation in expression patterns, then highlight species differences, both within and between islands.

### 3.1 Geographic variation in opsin expression

In support of our first prediction, both species expressed more SWS2b at Makobe Island (clear water) than at locations with higher turbidity (blue: *p* < 0.01 for all comparisons; red: *p* < 0.001; Figure 4). SWS2b expression at Anchor Island (also relatively clear water) was similar to that at the turbid locations (essentially zero). SWS2a expression did not differ between locations (*p* > 0.28). Geographic variation in RH2A and LWS expression was different between the two species; both opsins were influenced by significant island by species interactions (RH2A: *χ* ^2^(4) = 39.96, *p* < 0.001; LWS: *χ* ^2^(4) = 21.62, *p* = 0.0002). For the blue species, opsin expression profiles were similar between locations, except at clear water Makobe (Figure 4), where LWS expression was lower than at all other locations (*p* < 0.038 for all comparisons) and RH2A expression higher (vs. Python, Kissenda, Luanso: *p* < 0.029; Anchor and Makobe did not differ; *p* = 0.29). For the red species, LWS expression was lowest at Makobe, Python, and Kissenda, and highest at Anchor (vs. Python & Kissenda: *p* < 0.02; vs. Makobe: *p* = 0.052). RH2A expression was higher for the red species at turbid locations—Python and Kissenda—compared to locations with clearer water—Makobe (vs. Python: *p* = 0.018; vs. Kissenda: *p* = 0.090) and Anchor (*p* < 0.01 for both).

### 3.2 Species differences in opsin expression within islands

**Figure 5:**
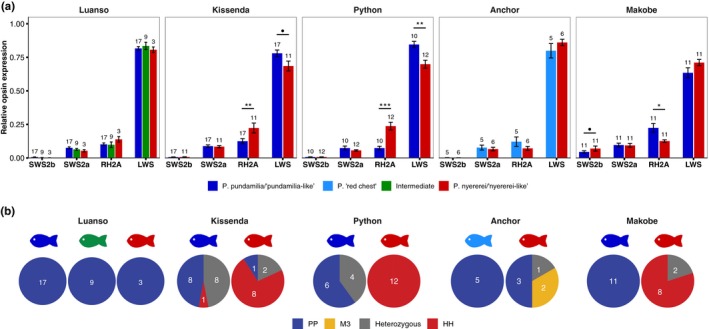
Island‐ and species‐specific opsin expression and LWS genotype. (a) Species differences in opsin expression varied across islands. *Makobe Island*: LWS expression did not differ between *Pundamilia pundamilia* and *Pundamilia nyererei* (*p* = 0.3) but RH2A expression was higher in *P. pundamilia* (*Z* = 3.02, *p* = 0.016). SWS2a did not differ (*p* > 0.9) and SWS2b was slightly higher in *P. nyererei* (*Z* = 2.63, *p* = 0.055). *Anchor Island*: all comparisons were nonsignificant (*p* > 0.86). *Python Island*: *P. *sp. “*pundamilia‐like*” expressed more LWS than *P. *sp. “*nyererei‐like*” (*Z* = 3.68, *p* < 0.01), while *P. *sp. “*nyererei‐like*” expressed more RH2A than *P. *sp. “*pundamilia‐like*” (*Z* = 5.00, *p* < 0.001). SWS2a or SWS2b expression did not differ (*p* > 0.83). *Kissenda Island*: LWS expression was slightly higher in *P. *sp. “*pundamilia‐like*” (*Z* = 2.64, *p* = 0.053) while *P. *sp. “*nyererei‐like*” expressed significantly more RH2A (*Z* = 3.32, *p* < 0.01). SWS2a and SWS2b expression did not differ (*p* > 0.9). *Luanso Island*: there were no differences in opsin expression (*p* > 0.9). Sample sizes are indicated above each bar and error bars represent ± standard error. ***indicates *p* < 0.001, **indicates *p* < 0.01, *indicates *p* < 0.05, • indicates *p* < 0.1. (b) Consistent with previously reported patterns (Seehausen et al., 2008), the blue species were generally “PP” genotypes and the red species were “HH” genotypes. Anchor Island had not been previously investigated: the “H” allele was absent, but the “M3” allele was present in the red phenotypes. All fish at Luanso Island were “PP” genotypes

Our second prediction was that, within islands, LWS expression would be higher in the red species and SWS2a/SWS2b expression would be higher in the blue species. Our results were partially in line with this prediction: LWS tended to be higher in the red types, but only at the islands with the clearest waters (Makobe and Anchor; Figure 5a). These are also the islands where the red species is *P. nyererei*. At the more turbid locations, Python and Kissenda, the blue species (*P. *sp. “*pundamilia‐like*”) tended to have higher LWS expression than the red species (*P. *sp. “*nyererei‐like*”). The reversed pattern was observed for RH2A expression (Figure 5a).

### 3.3 Light environment

To evaluate whether opsin expression profiles could be predicted by the specific light environment that the fish experience, our model included OR as an individual effect. OR had no influence in full models, nor when the species and island variables were removed (*p* > 0.41 for all opsins, see Table 1). It is possible that individual capture depth may not adequately represent the populations' light environment, so we also calculated population‐level OR scores using depth distribution data from a larger sample of fish (as reported in Seehausen et al., 2008) and reexamined the relationship between expression and OR. The results were quantitatively similar (see Table 1) and together indicate that the local light environment alone does not adequately predict variation in opsin expression. This is supported by the fact that similar light conditions occur in multiple habitats (island–depth combinations), yet we observed highly different opsin expression profiles (Figure S3).

### 3.4 Distribution of LWS genotypes

Consistent with prior work (Seehausen et al., 2008), color phenotype matched LWS genotype at Makobe and Python Islands: at these locations, most blue fish were “PP” genotypes and most red fish were “HH” genotypes (Figure 5b). We also observed a small number of heterozygotes (two red fish at Makobe; four blue fish at Python). A similar pattern was present at Kissenda, though we did observe one blue fish with the “HH” genotype, one red fish with the “PP” genotype, and a considerable number of heterozygotes (eight blue; two red). All fish at Luanso Island were “PP” genotypes (Figure 5b). LWS genotype had never been assessed at Anchor Island; we found that the blue species—*P. *“*red chest*”—had exclusively “PP” genotypes, while the red species—*P. nyererei*—had both “PP” and “M3” genotypes (Figure 5b). One red fish at Anchor was heterozygous. For LWS allele frequencies per species and location, see Table S3.

### 3.5 Relationship between LWS genotype and opsin expression

**Figure 6:**
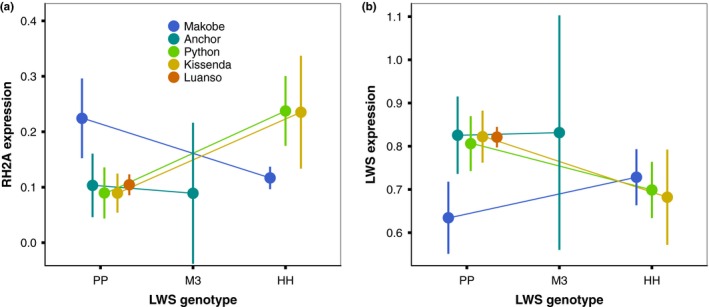
Relationship between LWS genotype and opsin expression differs between islands. Opsin expression is both genotype‐ and location‐dependent, as evidenced by the significant interaction of LWS genotype and island for RH2A (*χ* ^2^(5) = 41.24, *p* < 0.001) and LWS expression (*χ* ^2^(5) = 27.53, *p* < 0.001). In turbid waters (Python and Kissenda), individuals with LWS genotype “PP” had (a) lower RH2A expression and (b) higher LWS expression than individuals with “HH” genotypes. This pattern was reversed in clear waters (Makobe). All fish at Luanso Island were “PP” genotypes. Error bars represent ± 95% C.I

The data presented above suggest that there is no consistent relationship between LWS genotype and opsin expression across populations. Indeed, opsin expression significantly covaried with the interaction between LWS genotype and location (model: expression ~ genotype*island), for RH2A and LWS (*p* < 0.001 for both). As seen in Figure 6, in turbid waters (Python and Kissenda), individuals with LWS genotype “PP” had lower RH2A and higher LWS expression than individuals with “HH” genotypes. In clear water (Makobe), this pattern was reversed. For opsin expression patterns for each island and LWS genotype, see Figure S4.

### 3.6 Visual performance

If the variation reported above is adaptive, we predict that the observed combinations of opsin genotype and opsin expression maximize visual performance in the local environment. To test this prediction, we calculated the quantum catch (*Q_c_*) of the different genotypes from all locations (except Anchor—spectral measurements were unavailable), considering their opsin expression profiles. We then compared the *Q_c_* of the local fish to the *Q_c_* that would be achieved by hypothetical immigrants from other islands.

**Figure 7:**
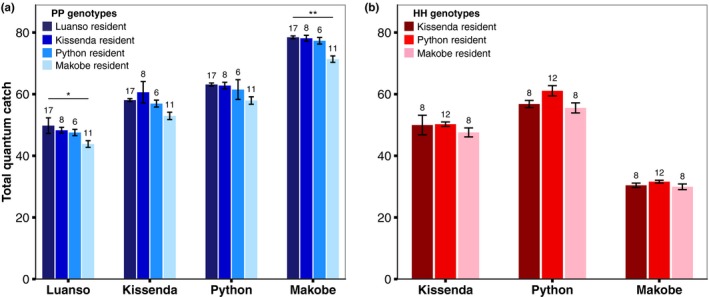
Similar visual performance despite different opsin expression profiles. For both the (a) blue and (b) red species, total *Q_c_* of the resident fish did not systematically differ from hypothetical immigrants. Here, all blue fish are “PP” genotypes and all red fish are “HH” genotypes. For *Q_c_* values of the individual opsins, see Figure S5. Sample sizes are indicated above each bar and error bars represent ±standard error. **indicates *p* < 0.01, *indicates *p* < 0.05

Despite geographic variation in opsin expression, visual performance did not consistently differ between the residents and hypothetical immigrants for either species (Figure 7). Only in one out of 18 comparisons did residents achieve significantly higher total *Q_c_*: the blue species at Luanso had higher total *Q_c_* than hypothetical immigrants from Makobe (all “PP” genotypes; *Z* = 3.76, *p* = 0.014). Within the red species (only “HH” genotypes), the resident populations never achieved higher total *Q_c_* than the transplants (Makobe: *p* > 0.9; Python: *p* > 0.12; Kissenda: *p* > 0.9). *Q_c_* values for the red species at Makobe were generally low, but all red fish from Makobe came from the lower end of the previously reported depth distribution (Seehausen et al., 2008), and thus from a narrow‐spectrum light environment. We therefore recalculated *Q_c_* for a more representative depth range, but this did not generate differences between residents and immigrants either (see Figure S6).

We also compared the visual performance of the heterozygous genotypes with the “HH” or “PP” genotypes (of the same color), within islands. We found no evidence of increased or decreased light capture in the heterozygotes (Figure S7). Thus, we cannot explain the presence or absence of heterozygous genotypes by visual system performance. Taken together, these results suggest that the observed combinations of opsin expression and LWS gene sequence do not maximize local visual performance.

## 4 DISCUSSION

Divergent adaptation to alternative visual habitats has been implicated in cichlid speciation. Previous work in *Pundamilia* has revealed correlations between the local light environment and LWS genotype across populations (Seehausen et al., 2008). The contribution of differential opsin expression to visual adaptation remained to be addressed: Haplochromine species and populations (in *Pundamilia* and other genera) differ in opsin expression (Carleton, 2009; Carleton et al., 2005; Hofmann et al., 2009, 2010; Parry et al., 2005; Smith et al., 2011; Spady et al., 2006) but a systematic exploration of this variation in *Pundamilia* was lacking. Here, we report that opsin expression profiles differ markedly between populations and do not covary with LWS genotype. Visual modeling suggests that the observed variation does not contribute to local visual performance.

### 4.1 High LWS expression

In general, we found high levels of LWS expression (~76%), followed by RH2A (~14%), SWS2a (~8%), and SWS2b (~2%). These results follow the previously reported expression patterns for Lake Victoria cichlids (Hofmann et al., 2009). They also match the general pattern in many other fish, where high LWS expression tends to occur in turbid environments (Ehlman et al., 2015; Lythgoe et al., 1994; Torres‐Dowdall et al., 2017). In line with this, the haplochromine cichlids from Lake Malawi (clear water) have lower LWS and higher SWS and RH2A expression than Lake Victoria *Pundamilia* (Carleton & Kocher, 2001; Hofmann et al., 2009; Smith et al., 2011).

### 4.2 Geographic variation in opsin expression

We predicted higher SWS2b expression at clear water locations (following: Hofmann et al., 2009). Our results conform to this pattern. At Makobe Island, where short‐wavelength light is more abundant than at the more turbid locations further south, SWS2b expression was high in both phenotypes. The waters at Anchor Island are also relatively clear but, in contrast to Makobe, SWS2b expression was low. The evolutionary history of this population has not been explored but, perhaps, it is similar to other Mwanza Gulf populations (Python/Kissenda), characterized by recent hybridization between the blue and red species (Meier et al., 2017, 2018) and low SWS2b expression. The other opsins did not show clinal variation in expression levels. SWS2a expression did not differ among locations, while RH2A and LWS were both species‐ and island‐specific. In the blue species, LWS expression was the lowest at clear water Makobe Island and RH2A expression was the highest. In the red species, LWS expression was less variable between islands but RH2A expression tended to be higher at more turbid locations (Python and Kissenda). These patterns could not be explained by variation in the local light environment alone—orange ratio (OR) did not predict opsin expression—but may be due to (a) the different evolutionary histories of the populations and/or (b) different modes of visual adaptation at different locations. We address both explanations in more detail below.

### 4.3 Species differences in opsin expression

Within each island, we predicted that the local red species would express more LWS and the blue species would express more SWS. We found inconsistent support for this prediction. SWS2a expression did not differ between the phenotypes at any of the five locations and SWS2b was slightly higher in *P. nyererei* at Makobe. Patterns of LWS and RH2A expression were more variable: at the two locations with clearer waters, Makobe and Anchor, the blue species (*P. pundamilia* and *P. *“*red chest*”) tended to express more RH2A and less LWS than the red species (*P. nyererei*). At locations with higher turbidity—Python and Kissenda Islands—the difference in expression pattern reversed: LWS expression was higher in *P. *sp. “*pundamilia‐like*” (significantly so at Python, trending at Kissenda) and RH2A expression was higher in *P. *sp. “*nyererei‐like*.” This is in opposition to our prediction but in agreement with results of Hofmann et al. (2009), who also observed higher LWS expression in the red types of the species pair *P. pundamilia* and *P. nyererei* from Senga Point (clear water) but higher LWS expression in the blue types for *P. *sp. “*pundamilia‐like*” and *P. *sp. “*nyererei‐like*” sampled at Kissenda and Python Island, respectively. Finally, at the most turbid location in our study, Luanso Island, we found no differentiation in opsin expression. This is consistent with the lack of genetic differentiation and overlapping depth ranges at this location, as documented previously (Meier et al., 2017; Seehausen et al., 2008).

Taken together, we find patterns of differentiation in opsin expression profiles at all studied locations where blue and red species are genetically differentiated, but not where they are not. The direction of differentiation between blue and red species, however, was opposite between the two sites with relatively clear waters versus the two sites with relatively turbid waters. This discrepancy may be related to the evolutionary histories of the populations. Meier et al. (2017) found that the most likely scenario for *Pundamilia* speciation involves divergence of *P. pundamilia* and *P. nyererei* outside the Mwanza Gulf, with settlement of both species at Makobe Island. *P. pundamilia* then colonized the Mwanza Gulf (including Python Island). Many generations later, this population received gene flow from *P. nyererei* leading to a renewed speciation event in which a “*nyererei‐like*” species with red males and a “*pundamilia‐like*” species with blue males emerged from the original *P. pundamilia* population at Python, within the past 500 years (Meier et al., 2017, 2018). This distinct evolutionary history (as well as possible mixing with other species in the Mwanza Gulf) may have resulted in different, better gulf‐adapted expression profiles. Inconsistent with this scenario, however, is that we did not find evidence for superior visual performance for the resident fish at Python and Kissenda—we discuss this more below.

### 4.4 Distribution of LWS genotypes

*Pundamilia* harbors three versions of the LWS opsin gene: The “H,” “P,” and recombinant “M3” alleles. At locations where the blue and red species are genetically differentiated—Makobe, Python, and Kissenda—the allele types were previously found to be nearly fixed in each species (Seehausen et al., 2008). We observed similar patterns; at Makobe and Python Islands, the “PP” genotype occurred only in the blue species and the “HH” genotype only in the red species. This pattern was also present at Kissenda, although we did observe two fish with mismatched genotypes (see Figure 5b). Heterozygous genotypes occurred at all three locations: Makobe (two red), Python (four blue), and Kissenda (eight blue, two red). At Anchor Island, which had not been previously investigated, all *P*. “*red chest*” were “PP” genotypes, and *P. nyererei* were “PP” or “M3” genotypes, plus one heterozygous individual. The “M3” allele also occurs at low frequency in *P. nyererei* at Makobe Island (Seehausen et al., 2008; not observed in the present sample) but our results suggest it may have replaced the “H” allele in red types at Anchor. This may reflect the evolutionary history of the Anchor population—perhaps Makobe *P. nyererei* with the “M3” allele colonized it—and/or the “M3” allele is selected for in the light environment at Anchor Island. Both of these scenarios are speculative and will require further study. Finally, at Luanso Island, all fish were “PP” genotypes, again consistent with earlier results and with the lack of genetic differentiation at this location (Meier et al., 2017; Seehausen et al., 2008). Our results confirm that LWS genotype is under divergent selection in blue versus red *Pundamilia* and that gene flow at this locus is more common at more turbid locations, where the species are less strongly isolated (both in space and in genome‐wide genetic variation).

### 4.5 Relationship between opsin expression and LWS genotype

Patterns of opsin expression did not consistently covary with species differentiation in LWS genotype. This was highlighted by significant interactions between *genotype* and *island* for both RH2A and LWS expression: the direction of differentiation in opsin expression profile between “HH” and “PP” genotypes at turbid Python and Kissenda Islands was the reverse of that at clear water Makobe (Figure 6). Estimates of visual performance, using Quantum catch calculations, suggest that this variation does not increase local visual performance: for both species, residents did not consistently achieve higher total light capture than hypothetical immigrants from other islands. Comparisons of the heterozygotes and “HH” or “PP” genotypes also revealed no difference in total light capture (see Figure S7), suggesting neither selection for nor against heterozygous genotypes.

Together, our results indicate that the observed combinations of opsin expression and LWS genotype do not maximize local visual performance. However, our estimates of visual performance may be inadequate. First, quantum catch is a relatively crude measure of visual perception, that may not reflect actual performance at relevant visual tasks in nature, such as object–background discrimination (Guthrie, 1986). Second, our visual model did not incorporate all aspects of visual perception. For example, cichlids can use either Vitamin A1‐ or Vitamin A2‐based chromophores (Torres‐Dowdall et al., 2017), which influences visual sensitivity (Dartnall & Lythgoe, 1965; Hárosi, 1994; Toyama et al., 2008). Chromophore usage has never been measured in *Pundamilia* but it might differ between populations and may contribute to visual adaptation. Prior work has estimated mixed chromophore usage in the red species (Carleton et al., 2005) but precise measurements are required to assess differences in visual performance across environments.

## 5 CONCLUSION

We analyzed opsin expression patterns of wild‐caught *Pundamilia* cichlids from several locations and depth ranges in Lake Victoria. Opsin expression differed between species and islands, and replicate populations of species pairs from clear waters were similar to each other but distinct from species pairs inhabiting turbid waters. These patterns could not be explained by variation in visual environments alone and did not consistently correlate with species differences in LWS opsin genotype. Visual modeling suggests that the observed combinations of opsin expression and LWS genotype do not maximize local visual performance. Our results highlight the need to explore other visual tuning mechanisms, as well as more sophisticated ways of measuring visual performance, to understand how different components of the visual system adapt and co‐evolve.

## CONFLICT OF INTEREST

None declared.

## AUTHOR CONTRIBUTIONS

MEM and DSW designed the study; DSW collected eye samples; RvE designed the qPCR protocol and created the standard construct; DSW and RM completed laboratory work, with assistance from RvE; WV established the sequencing protocol; OS confirmed fish identities from photographs and contributed field data. DSW performed the analyses, with assistance from MEM; DSW and MEM wrote the manuscript, with contributions from OS. All authors approved the contents of this manuscript.

## Data Availability

Data and R‐scripts are archived at:https://hdl.handle.net/10411/I1IUUQ. LWS sequences are available on GenBank (www.ncbi.nlm.nih.gov/genbank/), accession numbers: MN047529–MN047792.
