# Supplementary material for: Geographic variation in opsin expression does not align with opsin genotype in Lake Victoria cichlid populations
Source: Ecol Evol. 2019 Jul 9;9(15):8676–89. doi: 10.1002/ece3.5411 (PMC6686298; doi:10.1002/ece3.5411)
Supplement: Supplementary file 1 [file ECE3-9-8676-s001.pdf]

Supplemental Information

Geographic variation in opsin expression does not align with opsin genotype in Lake Victoria cichlid populations

Daniel Shane Wright, Roy Meijer, Roel van Eijk, Wicher Vos, Ole Seehausen, and Martine E. Maan

Calculating opsin mRNA expression – Previous studies (Carleton et al., 2005; Hofmann et al., 2009) calculated relative opsin expression as:

$$\frac{T_i}{T_{all}} = \frac{(1 + E_i)^{-Ct_i}}{\sum(1 + E_i)^{-Ct_i}}$$

where T_i/T_{all} is the relative gene expression, E_i is the PCR efficiency of each gene, and Ct_i is the critical threshold (or cycle number). PCR efficiency was determined from a construct containing all genes ligated together. Here, we also used a reference construct of all four opsin genes ligated together. However, we used linear regression to examine the relationship between Log(concentration) and *Ct* values of the construct, enabling us to calculate not only the slope (*m*) but also the intercept (*b*) of the regression. Using both these values, we calculated relative expression as:

$$\frac{N_{0i}}{N_{0all}} = \frac{exp^{\frac{(Ct_i - b)}{m}}}{\sum exp^{\frac{(Ct_i - b)}{m}}}$$

where N_{0i}/N_{0all} is the expression for a given opsin gene relative to the total expression of all measured opsin genes, Ct_i is the critical threshold value for the focal sample, and *b* and *m* are the intercept and slope values derived from the construct linear regression (also described in: Gallup, 2011).

SWS2b	Primer (F)	GCGCTGCACTTCCACCTC
	Primer (R)	GGCCACAGGAACACTGCAT
	Probe	FAM-TTGGATGGAGCAGGTATATCCCAGAGGG-TAMRA
SWS2a	Primer (F)	CAAGATYGAAGGTTTCATGGTA
	Primer (R)	CGCTCGAAAGCTATCACAGC
	Probe	FAM-ACTCGGTGGTATGGTAAGCCTGTGG-TAMRA
RH2A	Primer (F)	TTCTGTGCWATTGAGGATTC
	Primer(R)	CCAGGACAACAAGTGACCAGAG
	Probe	FAM-TGGCCACACTWGGAGGTGAAGTTGC-TAMRA
LWS	Primer (F)	CTGTGCTACCTTGCTGTGTGG
	Primer (R)	GCCTTCTGGGTTGACTCTGACT
	Probe	FAM-TGGCCATCCGTGCTGTTGC-TAMRA

Table S1. Gene specific primers and probes – Sequences of the primers/probes used in qPCR reactions.

Table S2. LWS genotyping – Sequencing results of the blue and red species at each island. All fish were sequenced in forward (F) and reverse (R) directions and allele type assigned from amino acid positions 216, 230, and 275 (Seehausen et al., 2008). Heterozygous genotypes (Het) had multiple peaks at one or more the polymorphic nucleotide sites (Fig. S2). Genomic DNA (gDNA) and complementary DNA (cDNA) were consistent for all homozygous genotypes. For heterozygotes, cDNA samples had multiple peaks at polymorphic sites, confirming expression of both alleles.

Makobe Island

P. nyererei

				nucleotide position				amino acid position			
**number**	**sample id**	**type**	**direction**	**647**	**688**	**823**	**824**	**216**	**230**	**275**	**allele**
49	105252	gDNA	F	A	G	T	G	Y	A	C	H
			R	A	G	T	G	Y	A	C	H
50	105250	gDNA	F	A	G	T	G	Y	A	C	H
			R	A	G	T	G	Y	A	C	H
52	105262	cDNA	F	A	G	T	G	Y	A	C	H
			R	A	G	T	G	Y	A	C	H
		gDNA	F	A	G	T	G	Y	A	C	H
			R	A	G	T	G	Y	A	C	H
53	105256	cDNA	F	A	G	T	G	Y	A	C	H
			R	A	G	T	G	Y	A	C	H
57	105249	gDNA	F	A	A (G)	A (T)	G (T)	Y	T	S	Het
			R	A	G (A)	A (T)	G (T)	Y	A	S	Het
103	105254	gDNA	F	A	G	T	G	Y	A	C	H
			R	A	G	T	G	Y	A	C	H
104	105257	gDNA	F	A	G	T	G	Y	A	C	H
			R	A	G	T	G	Y	A	C	H
106	105255	gDNA	F	A	A (G)	A (T)	G (T)	Y	T	S	Het
			R	A	G (A)	A (T)	G (T)	Y	A	S	Het
108	105258	gDNA	F	A	G	T	G	Y	A	C	H
			R	A	G	T	G	Y	A	C	H
113	105260	gDNA	F	A	G	T	G	Y	A	C	H
			R	A	G	T	G	Y	A	C	H

P. pundamilia

41	109251	gDNA	F	T	A	A	T	F	T	I	P
			R	T	A	A	T	F	T	I	P
67	106888	gDNA	F	T	A	A	T	F	T	I	P
			R	T	A	A	T	F	T	I	P
68	104394	gDNA	F	T	A	A	T	F	T	I	P
			R	T	A	A	T	F	T	I	P
73	106889	gDNA	F	T	A	A	T	F	T	I	P
			R	T	A	A	T	F	T	I	P
76	104396	gDNA	F	T	A	A	T	F	T	I	P
			R	T	A	A	T	F	T	I	P
114	106890	gDNA	F	T	A	A	T	F	T	I	P
			R	T	A	A	T	F	T	I	P
133	104397	gDNA	F	T	A	A	T	F	T	I	P
			R	T	A	A	T	F	T	I	P
137	109149	gDNA	F	T	A	A	T	F	T	I	P
			R	T	A	A	T	F	T	I	P
138	109148	gDNA	F	T	A	A	T	F	T	I	P
			R	T	A	A	T	F	T	I	P
186	109151	cDNA	F	T	A	A	T	F	T	I	P
			R	T	A	A	T	F	T	I	P
		gDNA	F	T	A	A	T	F	T	I	P
			R	T	A	A	T	F	T	I	P
187	104400	gDNA	F	T	A	A	T	F	T	I	P
			R	T	A	A	T	F	T	I	P

Anchor Island

P. nyererei

number	sample id	type	direction	nucleotide position 647	688	823	824	amino acid position 216	230	275	allele
121	105283	gDNA	F	T	A	A	T	F	T	I	P
			R	T	A	A	T	F	T	I	P
125	105285	cDNA	F	A (T)	T (A)	T (A)	G (T)	Y	S	C	Het
			R	A (T)	T (A)	A (T)	G (T)	Y	S	S	Het
169	105371	cDNA	F	A	A	A	T	Y	T	I	M3
			R	A	A	A	T	Y	T	I	M3
		gDNA	F	A	A	A	T	Y	T	I	M3
			R	A	A	A	T	Y	T	I	M3
173	105378	gDNA	F	T	A	A	T	F	T	I	P
			R	T	A	A	T	F	T	I	P
175	105381	cDNA	F	A	A	A	T	Y	T	I	M3
			R	A	A	A	T	Y	T	I	M3
		gDNA	F	A	A	A	T	Y	T	I	M3
			R	A	A	A	T	Y	T	I	M3
188	105377	gDNA	F	T	A	A	T	F	T	I	P
			R	T	A	A	T	F	T	I	P

P. 'red chest'

number	sample id	type	direction	647	688	823	824	216	230	275	allele
98	105281	gDNA	F	T	A	A	T	F	T	I	P
			R	T	A	A	T	F	T	I	P
100	105276	gDNA	F	T	A	A	T	F	T	I	P
			R	T	A	A	T	F	T	I	P
107	105280	gDNA	F	T	A	A	T	F	T	I	P
			R	T	A	A	T	F	T	I	P
109	105279	gDNA	F	T	A	A	T	F	T	I	P
			R	T	A	A	T	F	T	I	P
115	105284	gDNA	F	T	A	A	T	F	T	I	P
			R	T	A	A	T	F	T	I	P

Python Island

P. sp. 'nyererei-like'

number	sample id	type	direction	nucleotide position 647	688	823	824	amino acid position 216	230	275	allele
1	106470	gDNA	F	A	G	T	G	Y	A	C	H
			R	A	G	T	G	Y	A	C	H
7	106433	cDNA	F	A	G	T	G	Y	A	C	H
			R	A	G	T	G	Y	A	C	H
11	106435	gDNA	F	A	G	T	G	Y	A	C	H
			R	A	G	T	G	Y	A	C	H
17	106437	gDNA	F	A	G	T	G	Y	A	C	H
			R	A	G	T	G	Y	A	C	H
26	106427	cDNA	F	A	G	T	G	Y	A	C	H
			R	A	G	T	G	Y	A	C	H
27	106412	cDNA	F	A	G	T	G	Y	A	C	H
			R	A	G	T	G	Y	A	C	H
31	106414	cDNA	F	A	G	T	G	Y	A	C	H
			R	A	G	T	G	Y	A	C	H
		gDNA	F	A	G	T	G	Y	A	C	H
			R	A	G	T	G	Y	A	C	H
117	106422	gDNA	F	A	G	T	G	Y	A	C	H
			R	A	G	T	G	Y	A	C	H
122	106431	cDNA	F	A	G	T	G	Y	A	C	H
			R	A	G	T	G	Y	A	C	H
		gDNA	F	A	G	T	G	Y	A	C	H
			R	A	G	T	G	Y	A	C	H
126	106423	cDNA	F	A	G	T	G	Y	A	C	H
			R	A	G	T	G	Y	A	C	H
129	106418	cDNA	F	A	G	T	G	Y	A	C	H
			R	A	G	T	G	Y	A	C	H
146	106471	gDNA	F	A	G	T	G	Y	A	C	H
			R	A	G	T	G	Y	A	C	H

P. sp. 'pundamilia-like'

9	106452	gDNA	F	T	A	A	T	F	T	I	P
			R	T	A	A	T	F	T	I	P
16	106449	gDNA	F	T (A)	A (G)	A (T)	G (T)	F	T	S	Het
			R	T (A)	A (G)	A (T)	G (T)	F	T	S	Het
18	106451	cDNA	F	T	A	A	T	F	T	I	P
			R	T	A	A	T	F	T	I	P
38	103818	gDNA	F	T	A	A	T	F	T	I	P
			R	T	A	A	T	F	T	I	P
43	103829	gDNA	F	T (A)	A (G)	T (A)	G (T)	F	T	C	Het
			R	T (A)	G (A)	A (T)	G (T)	F	A	S	Het
131	106458	cDNA	F	A (T)	G (A)	T (A)	G (T)	Y	A	C	Het
			R	A (T)	G (A)	A (T)	T (G)	Y	A	I	Het
		gDNA	F	T (A)	A (G)	A (T)	G (T)	F	T	S	Het
			R	T (A)	A (G)	A (T)	G (T)	F	T	S	Het
132	106454	gDNA	F	T	A	A	T	F	T	I	P
			R	T	A	A	T	F	T	I	P
135	106445	gDNA	F	T	A	A	T	F	T	I	P
			R	T	A	A	T	F	T	I	P
136	106441	cDNA	F	T (A)	A (G)	T (A)	G (T)	F	T	C	Het
			R	A (T)	G (A)	A (T)	G (T)	Y	A	S	Het
		gDNA	F	T (A)	G (A)	A (T)	G (T)	F	A	S	Het
			R	T (A)	A (G)	A (T)	G (T)	F	T	S	Het
160	103828	gDNA	F	T	A	A	T	F	T	I	P
			R	T	A	A	T	F	T	I	P

Kissenda Island

P. sp. 'nyererei-like'

number	sample id	type	direction	nucleotide position				amino acid position			allele
				647	688	823	824	216	230	275	
2	105343	gDNA	F	A	G	T	G	Y	A	C	H
			R	A	G	T	G	Y	A	C	H
3	105342	gDNA	F	A	G	T	G	Y	A	C	H
			R	A	G	T	G	Y	A	C	H
58	104769	gDNA	F	A	G	T	G	Y	A	C	H
			R	A	G	T	G	Y	A	C	H
74	106088	gDNA	F	A	G	T	G	Y	A	C	H
			R	A	G	T	G	Y	A	C	H
80	106059	cDNA	F	A (T)	G (A)	T (A)	G (T)	Y	A	C	Het
			R	T (A)	A (G)	A (T)	G (T)	F	T	S	Het
84	106057	cDNA	F	A	G	T	G	Y	A	C	H
			R	A	G	T	G	Y	A	C	H
91	106061	cDNA	F	A	G	T	G	Y	A	C	H
			R	A	G	T	G	Y	A	C	H
94	106063	cDNA	F	A	G	T	G	Y	A	C	H
			R	A	G	T	G	Y	A	C	H
		gDNA	F	A	G	T	G	Y	A	C	H
			R	A	G	T	G	Y	A	C	H
140	105345	cDNA	F	A (T)	G (A)	T (A)	T (G)	Y	A	F	Het
			R	A (T)	G (A)	A (T)	G (T)	Y	A	S	Het
		gDNA	F	T (A)	A (G)	G (T)	T (G)	F	T	C	Het
			R	T (A)	G (A)	G (T)	T (G)	F	A	S	Het
142	105341	cDNA	F	A	G	T	G	Y	A	C	H
			R	A	G	T	G	Y	A	C	H
179	106070	cDNA	F	T	A	A	T	F	T	I	P
			R	T	A	A	T	F	T	I	P

P. sp. 'pundamilia-like'

number	sample id	type	direction	647	688	823	824	216	230	275	allele
75	106058	cDNA	F	T	A	A	T	F	T	I	P
			R	T	A	A	T	F	T	I	P
79	106084	cDNA	F	T (A)	G (A)	T (A)	T (G)	F	A	F	Het
			R	T (A)	G (A)	A (T)	G (T)	F	A	S	Het
		gDNA	F	T (A)	A (G)	A (T)	G (T)	F	T	S	Het
			R	T (A)	A (G)	A (T)	G (T)	F	T	S	Het
81	105360	gDNA	F	T	A	A	T	F	T	I	P
			R	T	A	A	T	F	T	I	P
83	106078	cDNA	F	T	A	A	T	F	T	I	P
			R	T	A	A	T	F	T	I	P
		gDNA	F	T	A	A	T	F	T	I	P
			R	T	A	A	T	F	T	I	P
87	106074	gDNA	F	T	A	A	T	F	T	I	P
			R	T	A	A	T	F	T	I	P
118	106087	cDNA	F	A	G (A)	T (A)	G (T)	Y	A	C	Het
			R	A	G (A)	A (T)	G (T)	Y	A	S	Het
		gDNA	F	A	A (G)	T (A)	G (T)	Y	T	C	Het
			R	A	G (A)	A (T)	G (T)	Y	A	S	Het
119	106077	cDNA	F	T (A)	G (A)	T (A)	G (T)	F	A	C	Het
			R	A (T)	G (A)	A (T)	G (T)	Y	A	S	Het
		gDNA	F	T (A)	A (G)	T (A)	G (T)	F	T	C	Het
			R	T (A)	G (A)	T (A)	G (T)	F	A	C	Het
124	106093	gDNA	F	A	G	T	G	Y	A	C	H
			R	A	G	T	G	Y	A	C	H
141	105346	cDNA	F	T (A)	A (G)	T (A)	G (T)	F	T	C	Het
			R	T (A)	G (A)	A (T)	G (T)	F	A	S	Het
		gDNA	F	T (A)	A (G)	A (T)	G (T)	F	T	S	Het
			R	T (A)	A (G)	A (T)	T (G)	F	T	I	Het
161	106065	cDNA	F	T	A	A	T	F	T	I	P
			R	T	A	A	T	F	T	I	P
163	106069	cDNA	F	T (A)	G (A)	T (A)	G (T)	F	A	C	Het
			R	A (T)	G (A)	A (T)	G (T)	Y	A	S	Het
		gDNA	F	T (A)	A (G)	A (T)	G (T)	F	T	S	Het
			R	T (A)	A (G)	A (T)	G (T)	F	T	S	Het

167	105353	gDNA	F	T	A	A	T	F	T	I	P
			R	T	A	A	T	F	T	I	P
177	106060	cDNA	F	T (A)	G (A)	T (A)	G (T)	F	A	C	Het
			R	A (T)	G (A)	A (T)	G (T)	Y	A	S	Het
		gDNA	F	T (A)	A (G)	A (T)	G (T)	F	T	S	Het
			R	T (A)	G (A)	A (T)	G (T)	F	A	S	Het
193	105355	gDNA	F	T	A	A	T	F	T	I	P
			R	T	A	A	T	F	T	I	P
196	106099	cDNA	F	T (A)	G (A)	T (A)	G (T)	F	A	C	Het
			R	T (A)	A (G)	A (T)	T (G)	F	T	I	Het
		gDNA	F	A (T)	A (G)	A (T)	G (T)	Y	T	S	Het
			R	T (A)	G (A)	A (T)	G (T)	F	A	S	Het
197	105356	cDNA	F	A (T)	G (A)	T (A)	G (T)	Y	A	C	Het
			R	A (T)	G (A)	A (T)	G (T)	Y	A	S	Het
		gDNA	F	T (A)	A (G)	A (T)	G (T)	F	T	S	Het
			R	T (A)	G (A)	A (A)	T (G)	F	A	I	Het

Luanso Island

Intermediate

number	sample id	type	direction	nucleotide position 647	688	823	824	amino acid position 216	230	275	allele
20	105866	gDNA	F	T	A	A	T	F	T	I	P
			R	T	A	A	T	F	T	I	P
21	105863	gDNA	F	T	A	A	T	F	T	I	P
			R	T	A	A	T	F	T	I	P
25	105881	gDNA	F	T	A	A	T	F	T	I	P
			R	T	A	A	T	F	T	I	P
51	103662	gDNA	F	T	A	A	T	F	T	I	P
			R	T	A	A	T	F	T	I	P
130	105877	cDNA	F	T	A	A	T	F	T	I	P
			R	T	A	A	T	F	T	I	P
144	103672	gDNA	F	T	A	A	T	F	T	I	P
			R	T	A	A	T	F	T	I	P
149	103667	cDNA	F	T	A	A	T	F	T	I	P
			R	T	A	A	T	F	T	I	P
		gDNA	F	T	A	A	T	F	T	I	P
			R	T	A	A	T	F	T	I	P
150	105864	cDNA	F	T	A	A	T	F	T	I	P
			R	T	A	A	T	F	T	I	P
172	105878	gDNA	F	T	A	A	T	F	T	I	P
			R	T	A	A	T	F	T	I	P

Reddish males

32	105885	gDNA	F	T	A	A	T	F	T	I	P
			R	T	A	A	T	F	T	I	P
155	105851	gDNA	F	T	A	A	T	F	T	I	P
			R	T	A	A	T	F	T	I	P
171	105879	cDNA	F	T	A	A	T	F	T	I	P
			R	T	A	A	T	F	T	I	P

Blueish males

23	105873	cDNA	F	T	A	A	T	F	T	I	P
			R	T	A	A	T	F	T	I	P
24	105875	cDNA	F	T	A	A	T	F	T	I	P
			R	T	A	A	T	F	T	I	P
30	105876	gDNA	F	T	A	A	T	F	T	I	P
			R	T	A	A	T	F	T	I	P
36	105850	gDNA	F	T	A	A	T	F	T	I	P
			R	T	A	A	T	F	T	I	P
48	103668	cDNA	F	T	A	A	T	F	T	I	P
			R	T	A	A	T	F	T	I	P
		gDNA	F	T	A	A	T	F	T	I	P
			R	T	A	A	T	F	T	I	P
60	105818	gDNA	F	T	A	A	T	F	T	I	P
			R	T	A	A	T	F	T	I	P
65	105827	gDNA	F	T	A	A	T	F	T	I	P
			R	T	A	A	T	F	T	I	P
101	105855	gDNA	F	T	A	A	T	F	T	I	P
			R	T	A	A	T	F	T	I	P
134	105859	gDNA	F	T	A	A	T	F	T	I	P
			R	T	A	A	T	F	T	I	P
147	105861	gDNA	F	T	A	A	T	F	T	I	P
			R	T	A	A	T	F	T	I	P
148	103669	cDNA	F	T	A	A	T	F	T	I	P
			R	T	A	A	T	F	T	I	P
		gDNA	F	T	A	A	T	F	T	I	P
			R	T	A	A	T	F	T	I	P
151	105857	gDNA	F	T	A	A	T	F	T	I	P
			R	T	A	A	T	F	T	I	P
152	103671	gDNA	F	T	A	A	T	F	T	I	P
			R	T	A	A	T	F	T	I	P
153	105854	gDNA	F	T	A	A	T	F	T	I	P

			R	T	A	A	T	F	T	I	P
156	105849	cDNA	F	T	A	A	T	F	T	I	P
			R	T	A	A	T	F	T	I	P
159	105847	gDNA	F	T	A	A	T	F	T	I	P
			R	T	A	A	T	F	T	I	P
178	105886	gDNA	F	T	A	A	T	F	T	I	P
			R	T	A	A	T	F	T	I	P

Species	Island	Colour	P	M3	H	Het	n†
P. luanso	Luanso	Blueish	100	0	0	0	34
P. luanso	Luanso	Intermediate	100	0	0	0	18
P. luanso	Luanso	Reddish	100	0	0	0	6
P. sp. 'pundamilia-like'	Kissenda	Blue	47	0	6	47	34
P. sp. 'nyererei-like'	Kissenda	Red	9	0	73	18	22
P. sp. 'pundamilia-like'	Python	Blue	60	0	0	40	20
P. sp. 'nyererei-like'	Python	Red	0	0	100	0	24
P. 'red chest'	Anchor	Blue	100	0	0	0	10
P. nyererei	Anchor	Red	50	33	0	17	12
P. pundamilia	Makobe	Blue	100	0	0	0	22
P. nyererei	Makobe	Red	0	0	80	20	20
Total							222

Table S3. LWS allele frequencies – The frequency (%) of LWS alleles for each sampled population. 'Het' represents sequences with multiple nucleotide calls at one or more of the nucleotide positions that differentiate H and P (see Fig. S3). n† indicates the number of sequences.

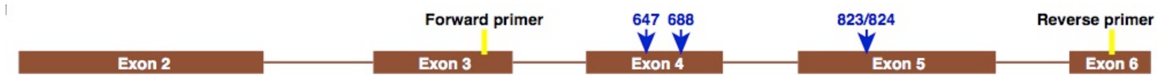

Figure S1. LWS sequencing design – We sequenced exons 4 and 5 of the LWS gene, to capture the known polymorphic nucleotide sites (in blue). Primers were located on exons 3 and 6.

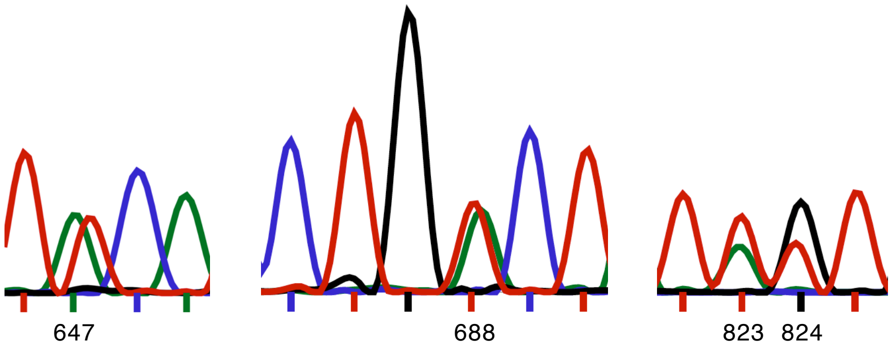

Figure S2. Heterozygous LWS genotypes – The sequencing results for some fish displayed multiple peaks at known polymorphic sites, shown here as the sequence chromatogram for nucleotide positions 647, 688 and 823/824.

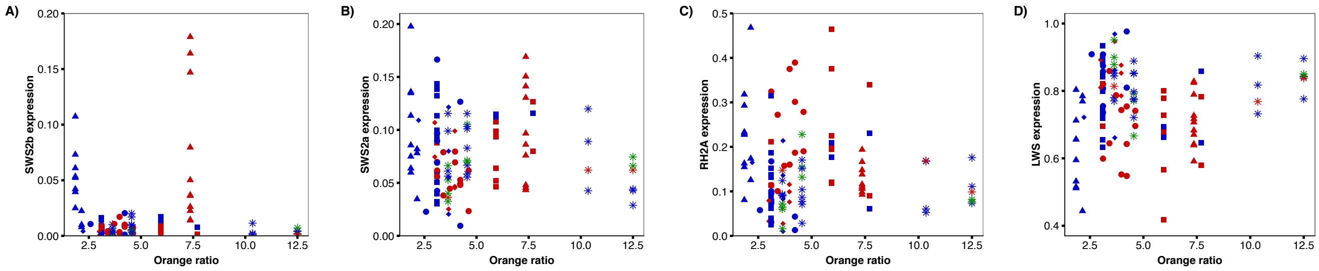

<u>Figure S3. Different opsin expression profiles in similar light environments</u> – Despite similar light conditions (OR values) for several sampled habitats across the Mwanza Gulf, opsin expression varied. Each symbol represents an individual fish; colours indicate phenotypes (blue, intermediate, red) and shapes represent islands (▲ Makobe, ◆ Anchor, ● Python, ■ Kissenda, ✱ Luanso).

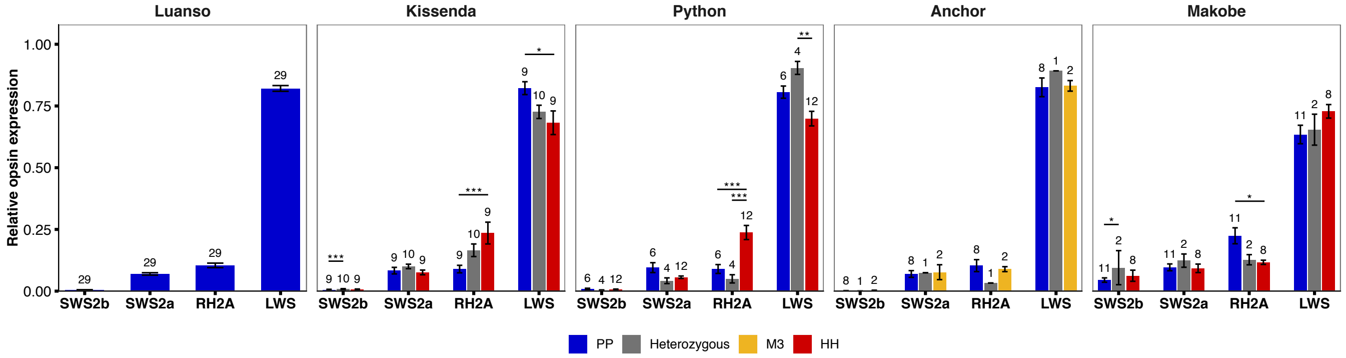

<u>Figure S4. Opsin expression for each genotype, separated by island</u> – Differences in opsin expression between LWS genotypes varied across islands. Sample sizes are indicated above each bar and error bars represent ± standard error. ***indicates $P < 0.001$, **indicates $P < 0.01$, *indicates $P < 0.05$, • indicates $P < 0.1$.

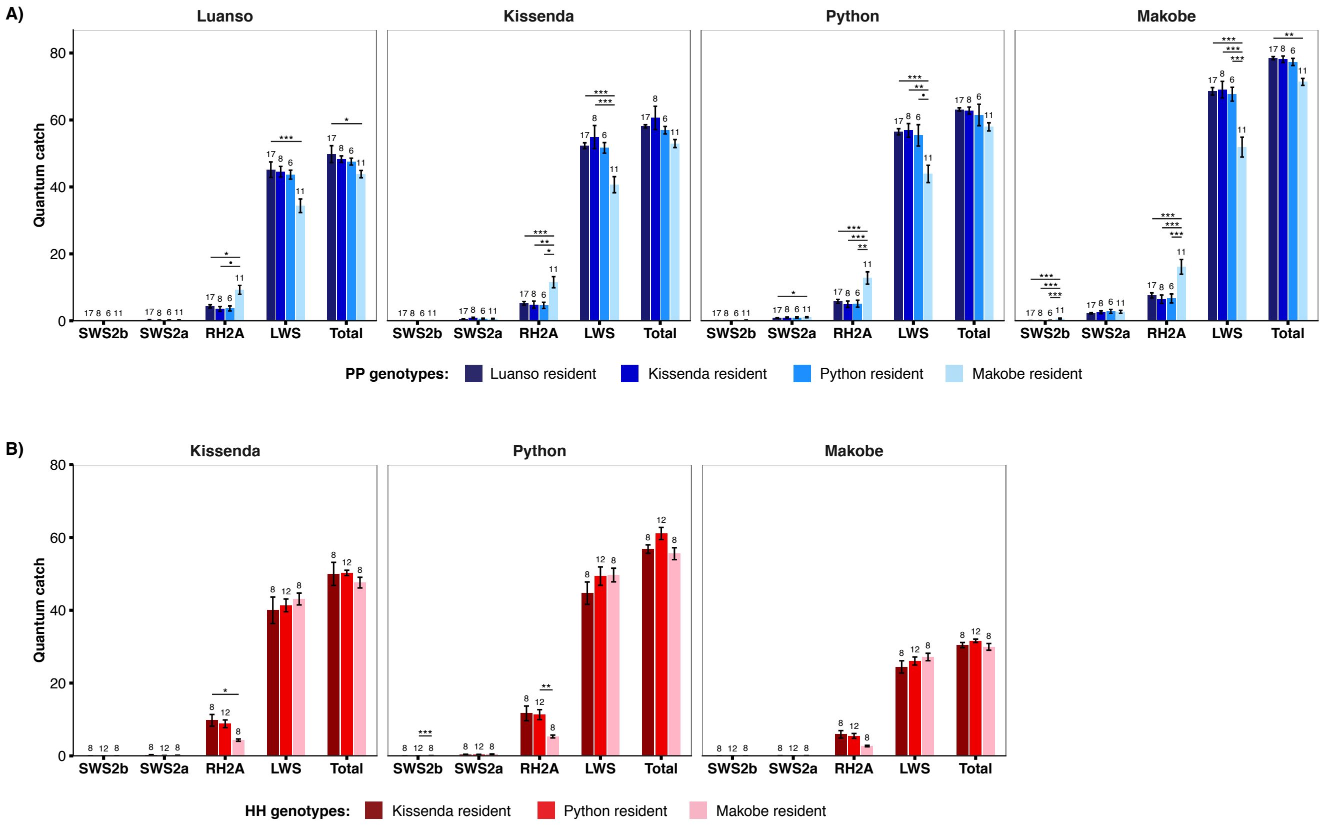

<u>Figure S5. Similar visual performance despite different opsin expression profiles</u> – Quantum catch (Qc) estimates for each opsin, comparing residents and hypothetical immigrants from other locations. For both the (A) blue and (B) red species, total Qc of the resident fish did not systematically differ from hypothetical immigrants. Here, all blue fish are 'PP' genotypes and all red fish are 'HH' genotypes. Sample sizes are indicated above each bar and error bars represent ± standard error. ***indicates $P < 0.001$, **indicates $P < 0.01$, *indicates $P < 0.05$, • indicates $P < 0.1$.

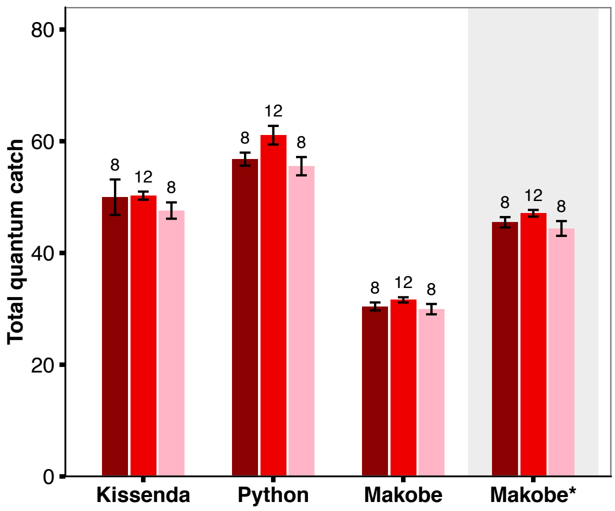

<u>Figure S6. Qc estimates at Makobe not due to sampling effort</u> – Qc estimates for the red species ('HH' genotypes) at Kissenda, Python and Makobe, calculated using the light spectra for individual capture depth (same data as in figure 7). Makobe* indicates Qc values recalculated based on the frequency-weighted mean depth of red males at Makobe Island (~6 meters; as reported in Seehausen et al., 2008). Qc estimates were higher, but still did not differ between residents and hypothetical immigrants.

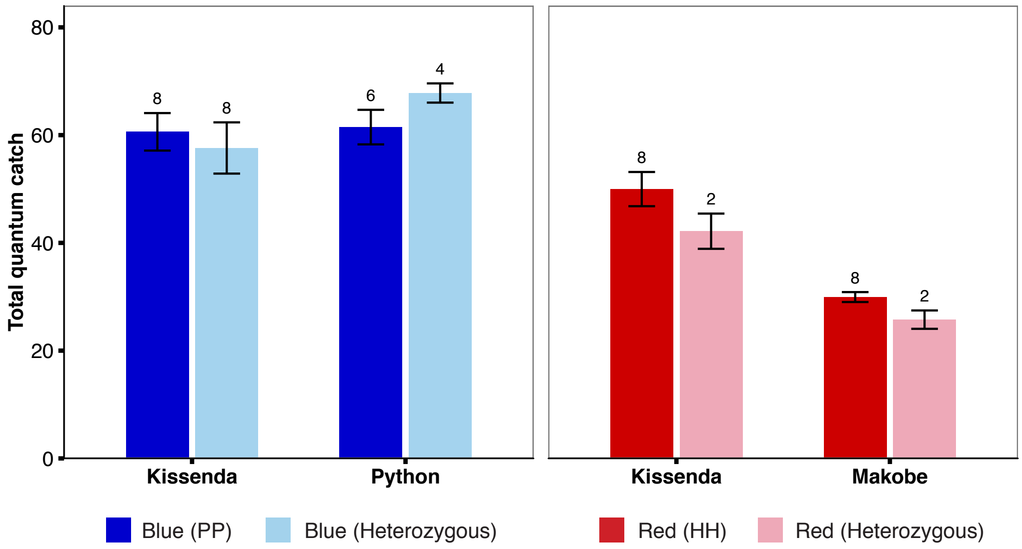

<u>Figure S7. Visual performance of heterozygous genotypes does not differ from homozygotes</u> – Heterozygous genotypes did not perform better or worse than the 'HH' or 'PP' genotypes (of the same colour) at any location. Sample sizes are indicated above each bar and error bars represent ± standard error.

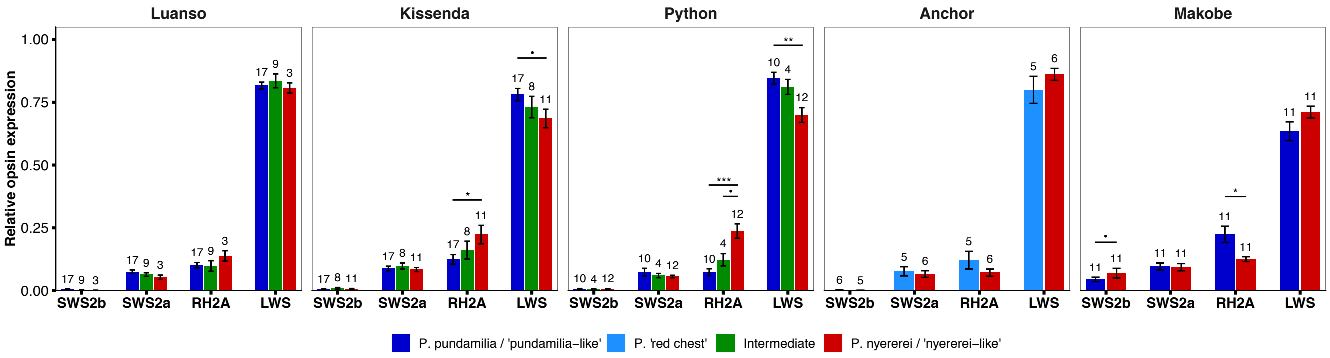

<u>Figure S8. Within-island, between-species variation in opsin expression, including intermediate phenotypes</u> – Species differences in opsin expression varied across islands. Intermediates at Python and Kissenda Island are based on morphological classification (and by colour scores at Luanso Island). Sample sizes are indicated above each bar and error bars represent ± standard error. ***indicates P < 0.001, **indicates P < 0.01, *indicates P < 0.05, • indicates P < 0.1.
